# Evolutionarily stable anti-cancer therapies by autologous cell defection

**DOI:** 10.1093/emph/eot014

**Published:** 2013-07-16

**Authors:** Marco Archetti

**Affiliations:** School of Biological Sciences, University of East Anglia, Norwich Research Park, Norwich NR4 7TJ, UK

## Abstract

Game theory suggests an anti-cancer treatment based on the use of modified cancer cells that disrupt cooperation within the tumor. Cancer cells are harvested from the patient, the genes for the production of essential growth factors are knocked out *in vitro* and the cells are then reinserted in the tumor, where they lead to its collapse.

**Background and objectives:** Current anti-cancer drugs and treatments based on gene therapy are prone to the evolution of resistance, because cancer is a process of clonal selection: resistant cell lines have a selective advantage and therefore increase in frequency, eventually conferring resistance to the whole tumor and leading to relapse. An effective treatment must be evolutionarily stable, that is, immune to the invasion of resistant mutant cells. This study shows how such a treatment can be achieved by autologous cell therapy using modified cancer cells, knocked out for genes coding for diffusible factors like growth factors.

**Methodology:** The evolutionary dynamics of a population of cells producing diffusible factors are analyzed using a nonlinear public goods game in a structured population in which the interaction neighborhood and the update neighborhood are decoupled. The analysis of the dynamics of the system reveals what interventions can drive the population to a stable equilibrium in which no diffusible factors are produced.

**Results:** A treatment based on autologous knockout cell therapy can be designed to lead to the spontaneous collapse of a tumor, without targeting directly the cancer cells, their growth factors or their receptors. Critical parameters that can make the therapy effective are identified. Concepts from evolutionary game theory and mechanism design, some of which are counterintuitive, can be adopted to optimize the treatment.

**Conclusions and implications:** Although it shares similarities with other approaches based on gene therapy and RNA interference, the method suggested here is evolutionarily stable under certain conditions. This method, named autologous cell defection, can be carried out using existing molecular biology and cell therapy techniques.

## Abstract (precis)

Game theory suggests an anti-cancer treatment based on the use of modified cancer cells that disrupt cooperation within the tumor. Cancer cells are harvested from the patient, the genes for the production of essential growth factors are knocked out in vitro and the cells are then reinserted in the tumor, where they lead to its collapse.

## BACKGROUND AND OBJECTIVES

The development of anti-cancer therapies normally begins with the identification of a molecule or pathway that is necessary for the development of the tumor and continues with the design of a method to target that molecule or pathway. Antiangiogenic therapies are a case in point [1]: it has long been known that oxygen concentration decreases with distance from a capillary [2, 3]; this led to the hypothesis that tumors cannot grow without inducing the formation of new blood vessels [4–6] and that disrupting neoangiogenesis could be an anti-cancer therapy [7]; the search for the ‘tumor angiogenesis factor’ lead to the identification of VEGF (vascular endothelial growth factor) as the primary responsible for neoangiogenesis [8, 9] and the eventual development of a monoclonal antibody targeting VEGF. A humanized variant of this anti-VEGF antibody led to the development of bevacizumab (Avastin; Genentech) [10], which was for a long time Roche’s best selling drug, with revenues in excess of 5 billion USD per year.

Unfortunately, even such a blockbuster drug can only extend survival for patients with certain types of cancer by a few months on average [11], far from being a cure for cancer. Overall mortality rates for cancer are still at levels comparable with half a century ago [12, 13], as chemotherapy, radiotherapy and surgery still account for the majority of treatments.

The problem with most current anti-cancer treatments is that cancer is a process of clonal selection within the body on the timescale of an individual’s lifetime [14–18], and mutant cell lines that are resistant to treatments can spread and eventually confer resistance to the whole tumor. This is why even the most modern anti-cancer drugs generally lead to relapse after few months, including modern drugs that, like Avastin, target growth factors [11, 19]. Gene therapy that uses small hairpin RNA (shRNA) to silence genes for growth factors faces similar problems and the expectations of RNA interference (RNAi) for anti-cancer treatments have been disappointing so far [20, 21]. The evolution of resistance is a problem for all current anti-cancer treatments, including modern approaches that, like antiangiogenic drugs and RNAi-based therapies, target growth factors. We need evolutionarily stable anti-cancer therapies. Little attention (if any), however, is devoted to understanding the evolutionary stability of treatments [22].

Here, we reverse the process of drug discovery by starting from an analysis of the dynamics of tumor development. Our scope is to identify conditions that would make a treatment stable against mutant cell lines; only then will we look at possible molecular tools to achieve the desired effect. Our starting point is therefore not the molecular biology of cancer, but the evolutionary dynamics of cancer. We focus on the production of growth factors by cancer cells, one of the hallmarks of cancer [23] and we start from the evolutionary game theory of growth factor production. More properly, the analysis is relevant to diffusible factors (including growth factors) that promote cell proliferation and survival.

Game theory is the branch of mathematics that studies strategic interactions, that is, interactions in which a player’s pay-off depends not only on his own decisions, but also on the other players' decision [24]. In the case of tumor progression, game theory is relevant to the study of growth factor production, because the growth factors produced by a cell diffuse and can be used by neighboring cells, raising a collective action problem; nonproducer cells can free-ride on the growth factors produced by their neighbors. Why then non-producing cells do not increase in frequency? These are typical issues studied by game theory. Although traditional game theory in economics assumes rational decisions and learning, in evolutionary game theory [25] rationality is replaced by the process of natural selection: the individuals that are programed to take the best ‘decision’ leave more progeny and increase in frequency within the population. Evolutionary game theory can help us understand the dynamics of diffusible factor production and identify which states of the population (of cancer cells) are stable under which conditions.

We can then go one step further and ask what can be done to change these dynamics; that is, we can study the mechanism design of anti-cancer therapies. Whereas game theory starts from a given problem (the game) and predicts the outcome, mechanism design can be thought of as a reverse game theory. The question is: how should we change the rules of the game in order to achieve the desired outcome? Mechanism design is traditionally used in Economics [26], for example, to design auctions and contracts or to understand what an institution can do in order to induce selfish individuals to contribute to a public good. Mechanism design in medicine needs to achieve the opposite: what should a treatment do in order to impair the production of diffusible factors by tumor cells?

What modern cancer treatments try to achieve, targeting growth factors, their genes or their receptors, is equivalent to reducing the availability of a public good. Although this may seem, at first sight, a rational strategy if one wants to reduce the growth of a tumor, we will show that this is not necessarily the case and we will describe different methods that can lead to an evolutionarily stable therapy. The method proposed here relies on autologous cell therapy: cells are harvested from the patient and genes coding for diffusible factors are knocked out; these modified cancer cells are then reintroduced in the tumor in order to modify the dynamics of the production of the factors coded by the knockout genes. Our scope is to show that, for certain parameters, such treatment is evolutionarily stable, that is, immune to the invasion of resistant cell lines.

## METHODOLOGY

We use a public goods game in the framework of evolutionary game theory. A cell can be a producer (+/+) or a nonproducer (—/—) of a diffusible factor. Producers pay a cost *c* that nonproducers do not pay (0 < *c* < 1). A cell (producer or nonproducer) benefits from the diffusible factors produced by all the cells in its group (of size *n*). The benefit *b*(*j*) for a cell is given by the logistic function *V*(*j*) = 1/[1 + *e*^-^*^s^*^(^*^j-k^*^)/^ *^n^*] of the number *j* of +/+ cells among the other cells (apart from self) in the group, normalized using a standard normalization [27]: *b*(*j*) = [*V*(*j*)-*V*(0)]/[*V*(*n*)-*V*(0)]. Using a logistic curve implies that, as is typical for biological molecules, including growth factors produced by cancer cells, the benefit has a sigmoid shape [28–30], with a synergistic increase for *j* < *k* and diminishing returns for *j* > *k, *where *k* is the inflection point of the benefit function (it is useful to define *h* = *k*/*n*); the parameter *s* controls the steepness of the function at the inflection point. Although the logistic function is a typical sigmoid function, we do not limit the analysis to a specific benefit function, but allow many possible shapes: *k→n* gives strictly increasing returns and *k→*0 strictly diminishing returns, whereas *s→∞* models a threshold PGG and *s→*0 models linear benefit (the N-person prisoner’s dilemma).

In an infinite, well-mixed population, the pay-offs of producers and of nonproducers are given by

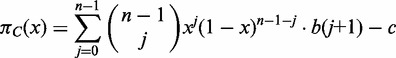


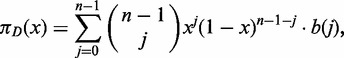

respectively, where 0 ≤ *x* ≤ 1 is the fraction of producers in the population, as a producer pays a cost *c* that a nonproducer does not pay, but its group has one more contributor (itself). The replicator dynamics is given by

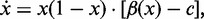

where the pay-off difference 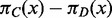 is written in the form 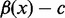 and

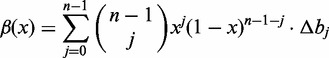

Although analyzing the gradient of selection of the replicator dynamics for well-mixed populations helps understand the logic of the problem [30], a realistic analysis of the dynamics of diffusible factors within a tumor must resort to a model of interactions in a spatially structured population.

A spatially structured population is modeled here as a two-dimensional regular lattice obtained using a modification of the GridGraph implementation in Mathematica version 8.0 (Wolfram Research Inc.) connecting opposing edges to form a toroidal network, in order to avoid edge effects. As in the standard approach, individuals occupy the nodes of the network (population size is fixed at 900) and social interactions proceed along the edges connecting the nodes. Different from the standard approach, however (in which an individual’s group is limited to her one-step neighbors and an individual plays multiple games centered on each of her neighbors [31]), here there is no reason to assume that the diffusion range of the public good is limited to a cell’s one-step neighbors. The interaction neighborhood and the update neighborhood are therefore decoupled: a cell’s group (of size *n*) is not limited to her one-step neighbors but is defined by the diffusion range (*d*) of the diffusible factor, that is, the number of edges between the focal cell and the most distant cell whose contribution affects the fitness of the focal cell. A cell’s pay-off is a function of the amount of factor produced by the group she belongs to. The process starts with a number of nonproducer cells placed on the graph; at each round a cell *x* with a pay-off *P_x_* is selected (at random) for update (death); in a deterministic approach, the neighboring cell with the highest pay-off will replace *x*. In a stochastic approach, a cell *y* (with a pay-off *P_y_*) is chosen among *x’*s neighbors. If *P_x_* > *P_y_*, no update occurs, whereas if *P_x_* < *P_y_*, *x* will adopt *y*’s strategy with a probability given by (*P_y_* - *P_x_*)/ *M*, where *M* ensures the proper normalization and is given by the maximum possible difference between the pay-offs of *x* and *y* [31]. Results are obtained averaging the final 200 of 1000 generations per cell, averaged over 10 different runs.

## RESULTS

**Figure 1.**
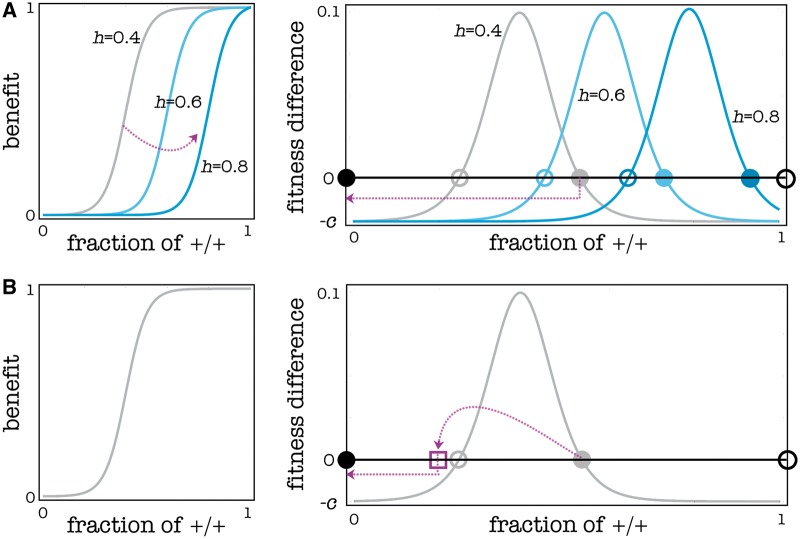
Difference between therapies that target growth factors and autologous cell therapy. (**A**) Targeting diffusible factors directly increases the threshold (*h*) of the PGG; as a consequence the system has new internal equilibria (empty circle: unstable; filled circle: stable). The therapy is successful (dotted line: the +/+ cells go extinct) only if the new unstable equilibrium (dark blue; *h* = 0.8) is above the previous stable equilibrium (gray; *h* = 0.4); if this is not the case the system will move to the new internal equilibrium (light blue; *h* = 0.6). (**B**) Autologous cell therapy does not rely on changing the benefit function of the diffusible factor, but must introduce a critical amount of −/− cells in order to destabilize the equilibrium and move the fraction of +/+ below the unstable equilibrium, after which the +/+ cells will go extinct. In all cases, the dynamics assume a well-mixed population with *n* = 50, *s* = 10, *h* = 0.4

A tumor can be thought of as a population of individuals facing a collective action problem for the production of a public good. Consider a population of cells (+/+) that produce a growth factor. If a mutant cell arises (—/—) that does not produce the growth factor, that cell and its descendants will still be able to use the growth factors produced by the surrounding +/+ cells. Such a PGG can have two types of equilibria (Fig. 1). In the first type of equilibrium, the +/+ cells and the −/− cells coexist. Heterogeneity of cells within a tumor is actually well documented for many types of cancers and many distinguishable phenotypes [32]. When one reduces the amount of a growth factor either by making it less available (using drugs like Avastin) or by targeting its gene product (using RNAi), the immediate result is, as expected, a sudden reduction in tumor growth. At the same time, however, one increases the amount of growth factors that must be produced, that is, the threshold necessary for the population to grow (because some of that growth factor is degraded or made unavailable by the treatment), thereby increasing the equilibrium frequency of +/+ cells. Unless the current (pre-treatment) equilibrium is below the new (post-treatment) unstable equilibrium, the population will adjust to the new conditions and reach a new stable equilibrium (Fig. 1).

**Figure 2.**
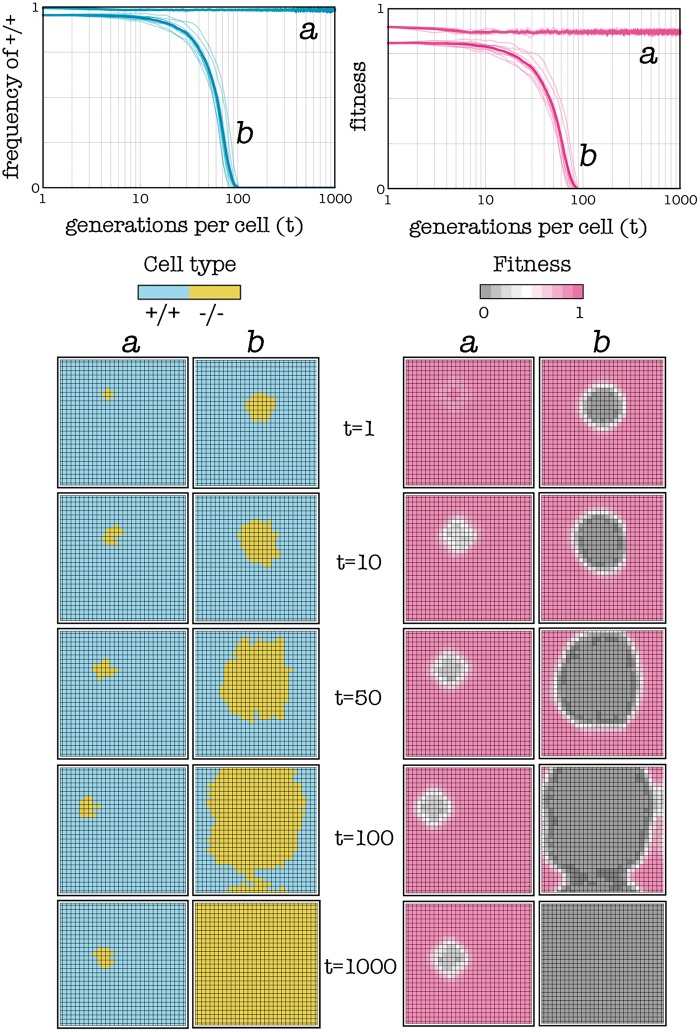
Introducing a critical amount of −/− cells can lead the population to collapse. The plots show frequencies and fitness over time (the bold line is the average of 10 simulations) and the lattices show snapshots of the population at different times. If the initial fraction of +/+ cells is locally below the unstable internal equilibrium (case b), clonal selection will spontaneously lead to the increase in frequency of −/− cells and to the consequent collapse of the tumor for lack of essential diffusible factors; if not (case a), the original equilibrium frequencies will persist. Stochastic update, *s* = 20, *h* = 0.7, *c* = 0.1, *d* = 5

Beside the mixed equilibrium in which +/+ and —/— cells coexist, PGGs usually have another type of equilibrium, in which the —/— cells replace entirely the +/+ cells (Fig. 1). This is what we want to achieve. Under certain conditions a population will evolve spontaneously to this latter equilibrium. What we want to do is create such conditions and let the populations spontaneously evolve to the equilibrium. In the simplest case, the correct conditions can be achieved simply by introducing in the tumor enough —/— cells (Fig. 2).

**Figure 3.**
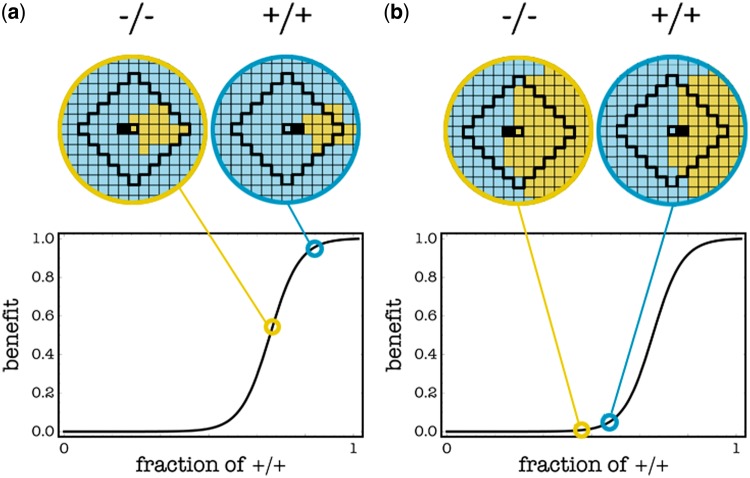
Details of a part of the population in Fig. 2 at *t* = 50 show the difference between the case in which the fraction of +/+ cells is locally above (**a**) or below (**b**) the internal unstable equilibrium. When a cell (black) dies, the adjacent cells (thick edges) compete to replace that cell’s node (only two competing cells are shown here); a −/− cell (yellow) does not pay the cost of producing diffusible factors, but is surrounded by more −/− cells than a +/+ cell (blue); in case a, the advantage in benefit for a +/+ cell is large enough to offset the cost (here *c* = 0.1); in case b, this advantage is not large enough. Only in case b will the population evolve to the pure −/− equilibrium. Stochastic update, *s* = 20, *h* = 0.7, *c* = 0.1, *d* = 5

**Figure 4.**
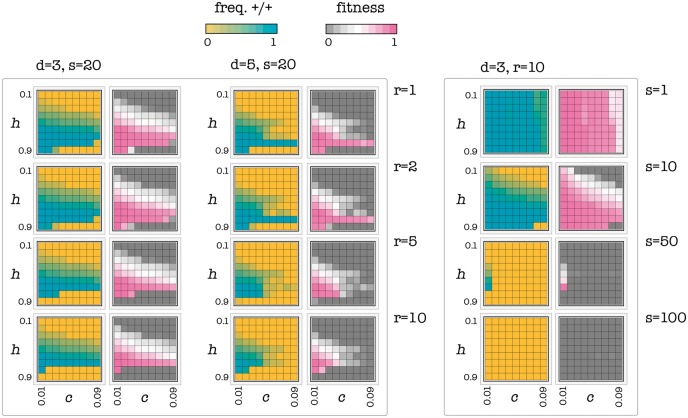
Effect of cost, type of benefit and number of defective cells. The color of each square in each plot represents the equilibrium values (frequency of +/+ or fitness) as a function of *h* (the threshold of the benefit function) and *c *(the cost/benefit ratio of producing the growth factor), when a group of −/− cells with radius *r* is introduced in the population. A large diffusion range (high *d*), a steep benefit function (high *s*), a large cost (*c*) and a larger initial amount of −/− cells (large *r*) are more likely to lead to the extinction of the +/+ cells. Deterministic update

The critical amount of —/— cells depends on the number of diffusible factors that have been knocked out (i.e. the relative cost of producing the growth factors), on the number of cells within their diffusion range (i.e. group size) and on the shape of the benefit function (i.e. *h* and *s*). A —/— cell has a selective advantage over a +/+ cell if the number of +/+ cells in the group is far from *k*, such that the difference in benefit between a +/+ and a —/— cell is lower than the cost paid by the +/+ cell (Fig. 3). In a well-mixed population the frequency of +/+ cells declines to zero only if it is below the unstable internal threshold (Fig. 1), whereas for higher frequencies of +/+, the population converges to the internal stable equilibrium, because the advantage of the —/— type declines as the frequency of +/+ type decline. In a spatially structured population, however, frequencies change only locally, within groups at the interface between +/+ and —/— cells, because these groups change in position as the —/— cells replace the +/+ cells. In a spatially structured population, —/— cells can go to fixation even for values of the threshold that in a well-mixed population would lead to a stable coexistence of +/+ and —/—. Under certain conditions, however, even if one introduces a large number of —/— cells, the system can evolve to a stable equilibrium in which +/+ persist (Fig. 4). In these cases, one must adopt additional strategies to achieve conditions that are conductive to the desired dynamics and equilibrium (the extinction of +/+ cells).

**Figure 5.**
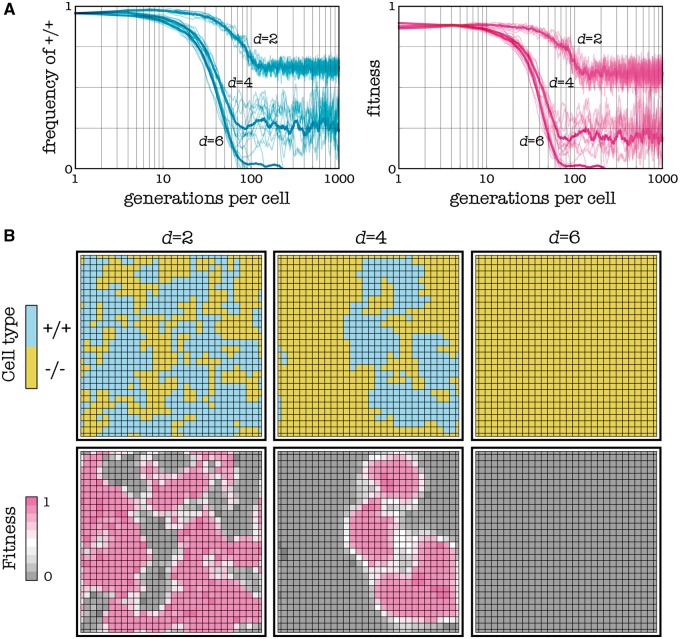
Importance of the diffusion range. The plots show frequencies and fitness over time (the bold line is the average of 10 simulations) and the lattices show the population after 1000 generations per cell. If the diffusible factor has a short diffusion range (*d*) −/− cells and +/+ cells can coexist under conditions that, with a larger diffusion range, will lead to the extinction of the +/+ cells. Deterministic update, *s* = 20, *h* = 0.5, *c* = 0.1

The first approach we can use is to extend the diffusion range of the diffusible factor. This is equivalent to increasing the number of cells that benefit from the production of a cell’s diffusible factors. We know from the theory (Fig. 5; see also [27]) that the provision of diffusible factors is less efficient in larger groups and that if group size is large enough, cooperation collapses. The diffusion range of the diffusible factor may be extended in a number of ways: by disrupting the binding molecules on the extra-cellular matrix or the binding domains on the diffusible factors; by adding soluble binding domains to saturate the binding molecules on the extra-cellular matrix or the binding domains on the diffusible factors or by increasing the amount of long-range isoforms of the factors. What these binding molecules and isoforms are, and more in general how to achieve this, depends on the type of tumor and diffusible factor. It is important to point out that the amount and efficacy of circulating diffusible factors and of their receptors remains the same, because one cell’s factors diffuse further away, but that cell receives additional factors from other cells, whose factors also diffuse further. The evolutionary response of the population, however, changes because group size changes and it can lead to the (evolutionarily stable) equilibrium in which all cells are —/— (Fig. 5).

**Figure 6.**
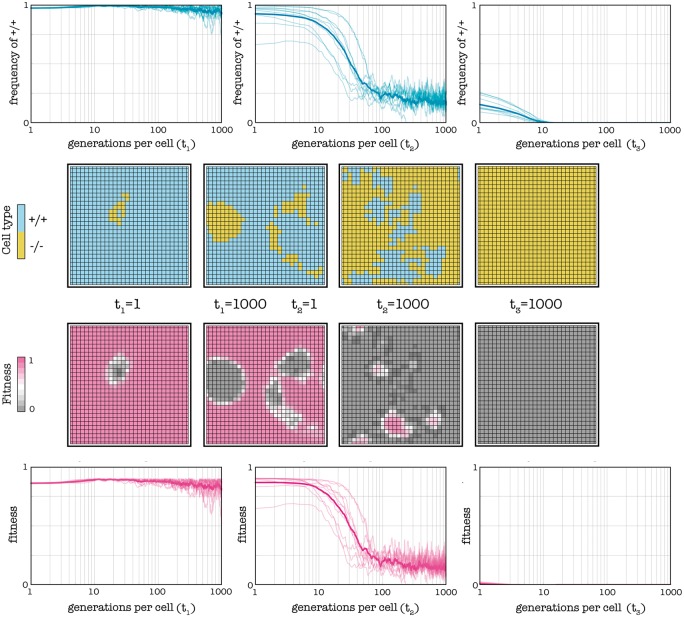
Temporarily increasing the amount of diffusible factors. The plots show frequencies and fitness over time and the lattices show snapshots of the population at different times. By providing exogenous diffusible factors (at *t*_2_ = 1) the threshold (*h* = 0.7) decreases (to *h* = 0.3) and, as a consequence, the frequency of +/+ cells declines toward a new stable internal equilibrium. When the provision of exogenous diffusible factors is interrupted (at *t*_3_ = 1), the threshold returns to the original value (*h* = 0.7); because the fraction of +/+ is now below the new (equal to the original) unstable equilibrium, +/+ cells will go extinct. Deterministic update, *s* = 20, *c* = 0.1, *d* = 3

Another possible enhancement is the temporary provision of exogenous diffusible factors (Fig. 6). This seems the opposite of what a drug should do. The logic here is to increase the cost/benefit ratio of producing endogenous diffusible factor by reducing the relative benefit of its production (or, in other words, to reduce the threshold of +/+ cells necessary for the diffusible factor to produce a given benefit). Even though the amount of available diffusible factor increases temporarily, growth rates will not increase much because of diminishing returns (assuming the benefit of diffusible factors is a sigmoid function of its concentration) and even if growth rates may suddenly increase, the crucial point is that the fraction of +/+ cells will immediately start to decrease and reach a new equilibrium. Our goal is to make this new equilibrium lower than the original unstable equilibrium. At this point, the system will be in the domain of attraction of the equilibrium in which all cells are —/— (Fig. 1), and when the external provision of diffusible factors is interrupted, the fraction of +/+ cells will decline to zero (Fig. 6).

## CONCLUSIONS AND IMPLICATIONS

The method suggested here can be considered a type of gene therapy, harvesting autologous cancer cells from a patient and genetically modifying them *in vitro* (knocking out genes coding for diffusible factors, rather than adding genes) before reinserting them *in vivo*. An appropriate name could be cell defection (for defective cells and for defector strategy).

The basic idea is to fight cancer using modified cancer cells that are defective for the production of essential diffusible factors. As these modified cells (—/—) do not produce the diffusible factors but can still use (at no cost) the factors produced by their neighbors, they have a replication advantage over the +/+ cells and will increase in frequency, like a tumor within the tumor. Eventually, the tumor will collapse (or slow down in growth) for lack of essential diffusible factors. In practice, our goal is to use modified cancer cells as free riders to induce a ‘tragedy of the commons’ [33] in the cancer population. In contrast to existing treatments, in which evolution (of resistance) is undesired, in our method the evolutionary response is what produces the desired effect; resistance cannot evolve because mutant cells that do produce diffusible factors (+/+) have a lower fitness in a population of —/— cells.

Directly targeting growth factors or their receptors is a typical approach of modern anti-cancer drugs (like Avastin). Although it has been suggested that attacking diffusible factors may be less susceptible to the evolution of resistance [34–36], the long-term failure of these drugs shows that this approach does not work as expected. One of the problems with therapies that target growth factors directly is that, as we have seen, when one reduces the amount of diffusible factor available, although the immediate result is a sudden reduction in tumor growth (because the threshold necessary to achieve the original benefit is not reached), the amount of diffusible factors necessary for the cells to achieve a certain benefit increases, which increase the equilibrium frequency of producers. Relapse, therefore, is due to the fact that the population adjusts to a new equilibrium. Complete suppression of circulating growth factors would produce the desired outcome, but is difficult to achieve.

Existing gene therapy approaches that target growth factors are likely to be unstable as well. Impairing proto-oncogenes using RNAi is unlikely to work, because it is prone to the evolution of resistance—not different from the effect of a drug that impairs the product of the proto-oncogenes. Restoring tumor suppressor genes (like p53) is unlikely to work in the long term because the modified cells have a private disadvantage against nonmodified cells. Existing gene therapy methods could only work if they were able to target all the cells in the tumor. It is not surprising that most of the efforts in the field of RNAi are devoted to the problem of efficient delivery [6].

An approach that seems more promising in the light of evolutionary dynamics is the use of shRNA to silence genes for growth factors. Although this looks similar to our method of using −/− cells, silenced cells (created *in vivo* by shRNA given systemically via a plasmid embedded in a delivery system) are different from knockout (−/−) cells because even though silenced cells do not produce the growth factors, they produce extra RNA and therefore, unlike our −/− cells, they will not have a selective advantage over the original cancer cells if the cost of extra shRNA overcomes the benefit of not producing the growth factor. Apart from the energetic costs of extra RNA production [37], which can overcome the benefits of lower protein production, the toxicity of shRNA due to off-target effects and interference with endogenous gene silencing (which would be even more pronounced with multiple knockdowns) is well known. This and other safety concerns have prevented further major developments with shRNA [6]. Knockout autologous cells do not have these toxicity and safety problems.

Autologous cell defection therapy is not without potential problems. Tumor cells often release diffusible factors that induce stromal cells (cancer-associated fibroblasts, endothelial cells, myofibroblasts and immune/inflammatory cells, including T- and B-cells, macrophages, neutrophils, mast cells, mesenchymal stem cells and other bone marrow-derived cells) to produce other diffusible factors and these stroma-produced factors also promote the proliferation of cancer cells [38–40]. A possible problem with the autologous cell therapy proposed here, therefore, might be that, even if −/− cells go to fixation within the tumor, diffusible factors provided by the stroma may still enable the tumor to grow. The stroma, however, produces diffusible factors that improve tumor fitness only when induced (‘activated’) to do so by signals released by the tumor itself. The ‘signals’ are diffusible factors themselves produced by the cancer cells (usually growth factors that may be the same or different from the diffusible factors released in response by the stromal cells). Their dynamics are the same as for diffusible factors that affect the fitness of tumor cells directly (without interactions with the stroma); indeed, stroma-produced diffusible factors are even more ‘public’ goods than the growth factors produced by the tumor cells (which will, with a certain degree, act as private goods for the producing cells). The fact that the stroma produces diffusible factors in response to signals from the tumor, therefore, does not seem to represent a serious obstacle to the use of autologous defective.

A more serious problem may arise if the tumor has mutations that make a diffusible factor receptor, or its downstream signal transduction pathway (that leads to the regulation of gene expression), constitutively active. In this case, the cancer cell is effectively independent from that diffusible factor. Such mutations are known to be responsible for the failure of drugs that target growth factor receptors like Herceptin and Erbitux [41]. Autologous defective cell therapy would be ineffective as well if the knockout process is limited to a single diffusible factor for which the cell has a constitutively active receptor (or signal transduction pathway). However, the (autologous) −/− cells will themselves have a constitutive receptor for that diffusible factor, and therefore, still have an advantage against +/+ cells due to the lack of production cost for the diffusible factor. More importantly, the use of −/− cells will still be effective if multiple diffusible factor genes are knocked out, unless the original +/+ cells are constitutively active for all diffusible factor receptors, which is unlikely. It is more likely that a downstream signal (like Ras) common to different receptors may be constitutively active. Such downstream signals, however, will have only a specific fitness effect (e.g. stimulate cell proliferation), whereas growth factors generally have multiple effects (e.g. cell proliferation, protection against apoptosis or against immune system reaction).

Autologous cell defection therapy is proposed here mainly as a method for treating primary, nonmetastatic tumors that can be directly accessed by cell injection, and seems inherently inadequate against metastatic cancer. One could speculate, however, that by injecting −/− cells systemically (through the bloodstream originating from the site of the primary tumor) it might be possible to reach the sites of the metastases and therefore even treat metastatic cancer. Clearly, injecting cancer cells systemically makes sense under the assumption that −/− cells are only able to grow in the presence of other +/+ cancer cells, that is, at sites of metastasis; here the −/− cells will have the same selective advantage and lead to the same effect described for primary tumors. Finally, the method relies on the fact that cells compete with each other, which may not be the case in the early stages of the tumor, during the initial growth of metastases or if cells do not divide rapidly.

Although the concept of autologous cell defection is grounded in evolutionary game theory, the path from the theory to actual medical applications is clearly a long one. Cancer dynamics is a much more complex process than the one described here [42]. In order to provide more precise estimates, the methods used here could be extended to take into account, for example, three-dimensional Voronoi graphs rather than regular two-dimensional lattices. The benefit of the diffusible factor produced by a cell should depend on the distance from the producing cell, on the type of tissue and diffusible factor and on the developmental stage of the tumor. Further theory and experimental validation of the method will be reported shortly.
